# Feasibility and acceptability of a midwife-led intervention programme called 'Eat Well Keep Active' to encourage a healthy lifestyle in pregnancy

**DOI:** 10.1186/1471-2393-12-27

**Published:** 2012-04-11

**Authors:** Lucie Warren, Jaynie Rance, Billie Hunter

**Affiliations:** 1 College of Human & Health Sciences, Swansea University, Swansea SA1 8PP, UK

**Keywords:** Behaviour change, Motivational interviewing, Pregnancy, Diet, Activity, Goal setting

## Abstract

**Background:**

Eating a diet that is high in fat and sugar and having a sedentary lifestyle during pregnancy is understood to increase the risk of excessive gestational weight gain and obesity following the birth of the baby. However, there are no clinical guidelines in the UK on what is considered to be appropriate gestational weight gain. Indeed, clinical recommendations discourage the routine re-weighing of pregnant women, stating instead that women should be advised regarding their diet and activity levels, in order to prevent excessive weight gain. Pregnancy is seen as a time when many women may have an increased motivation to improve their lifestyle behaviours for the benefit of the fetus. However, it is evident that many women have difficulty in both maintaining a healthy balanced diet and remaining active through pregnancy. It would seem that midwives may be ideally placed to assist women to make and maintain healthier lifestyle choices during pregnancy.

**Methods/design:**

This study will look at the feasibility and acceptability of a newly devised intervention programme called 'Eat Well Keep Active'. Participants will complete a questionnaire prior to the programme to obtain baseline data on food frequency, physical activity and to gauge their perception of personal ability to improve/maintain healthy lifestyle. The programme comprises client centred techniques; motivational interviewing and goal setting delivered early in pregnancy (12-16 weeks) with the aim of supporting a healthy well balanced diet and either continuing or commencing appropriate levels of physical activity. Participants will then be followed up six weeks following the intervention with a one-to-one interview, and a further brief questionnaire. The interview will provide preliminary data regarding perceived effectiveness and acceptability of the 'Eat Well Keep Active' programme whilst the questionnaire will provide data regarding changes in the confidence of participants to lead a healthy lifestyle.

**Discussion:**

There is an identified need for effective interventions that assist pregnant women in managing their diet and activity levels. Results from this study will demonstrate whether women find this programme of intervention, designed to elicit healthy behaviours in pregnancy, acceptable and whether they perceive it to be effective.

## Background

The topic of obesity has received much attention in recent years; specifically maternal obesity during pregnancy which has been of concern since the 2007 Confidential Enquiries into Maternal and Child Health identified that obese women and their babies were at increased risk of morbidity and mortality [1]. Pregnancy can be seen to be a key time in the development of obesity where if the old myths of eating for two and resting are adhered to, excessive weight gain is the likely result. It would appear that pregnancy is an ideal time in which to support and encourage women to make healthy appropriate choices regarding their diet and activity levels and in so doing reduce the risk of excessive gestational weight gain due to health behaviours.

Although there are no clinical guidelines in the United Kingdom regarding appropriate gestational weight gain, in the USA and parts of Europe guidelines for weight gain based on pre-pregnancy Body Mass Index are in use. The US Institute of Medicine guidelines were developed to reduce poor fetal and neonatal outcomes as a result of inadequate maternal diet [2]. Despite the establishment of the guidelines it is thought that as many as 20%-60% of pregnant women in the USA exceed the recommended weight gain [3-5]. Excessive weight gain in pregnancy is known to result in an increase in poor obstetric outcomes, similar to those found with maternal obesity, including: pre-eclampsia, hypertension, gestational diabetes, induction of labour, large for gestational age infants, caesarean section, and assisted delivery of infant [3,6]. It is understood that these women are more likely to remain obese, which in turn has implications for long-term health and future pregnancies [7,8].

There is also research to suggest that diet during pregnancy can influence offspring dietary habits and risk of obesity. Experimental animal studies have found that rats fed on an energy dense diet as opposed to a balanced diet during pregnancy, produce offspring with a greater preference for junk food and a greater propensity for obesity [9]. This finding suggests that the quality of the food eaten in pregnancy may in some way pre-programme offspring to over consume. On this basis women should be encouraged to have a healthy balanced diet in pregnancy, not just to improve their own health and provide the best start for the baby but also as a wider preventative strategy to reduce obesity in children.

Two UK documents were produced in 2010 regarding management of weight in pregnancy, both aimed at all clinicians involved in maternity care. Firstly the Centre for Maternal and Child Enquiries and the Royal College of Obstetricians and Gynaecologists developed a joint guideline on the management of obese pregnant women [10]. Alongside recommendations for specific clinical care of this high risk group of women, it instructed clinicians to alert women to the risks of obesity in pregnancy and the importance of a healthy diet and keeping active to reduce the risk of excessive weight gain and gestational diabetes. Secondly the National Institute for Health and Clinical Excellence produced public health guidance on the topic of weight management before, during and after pregnancy [11]. This too recommended that health professionals involved in pregnancy care should advise all women that it is beneficial to have a healthy diet and to keep physically active whilst pregnant and that doing so will assist them to achieve a healthy weight following the birth. This document also recognised the need to identify effective ways of helping women to manage weight during pregnancy.

Although the guidance regarding diet and exercise in both these documents is not new, it does give further support and clarification to clinicians regarding the importance of providing timely lifestyle advice to pregnant women.

Evidence suggests that, although weight and weight gain are influenced by a number of different factors such as genetics and environment, a key to weight control is individual motivation to limit calorie intake and increase physical activity [12], indeed, research [13-16] has identified that the dietary and lifestyle behaviours during pregnancy influence gestational weight gain.

A preliminary study conducted by the authors (not yet published) found that although participants frequently reported that they intended to eat a balanced and healthy diet, avoiding unhealthy snacks or meals and to commence regular exercise, many were unable to fulfil these goals. It would appear that the motivation which initially identified healthy lifestyle goals was unable to sustain this behaviour throughout the course of the pregnancy.

Self Determination Theory (SDT) is a psychological theory of human growth and development which focuses on motivation [17]. The theorists posit that the more self- determined an action is, the more stable the motivation and this results in increasing perseverance of behaviour. SDT provides a framework for improving the quality of motivation, through ensuring that the environment supports the individual to satisfy three fundamental psychological needs: autonomy, competence and relatedness.

It is evident that excessive weight gain in pregnancy increases the risk of obstetric, maternal, fetal and neonatal complications. It would appear that pregnancy is an ideal time to address the issue of preventing and/or controlling obesity, as women are routinely accessing healthcare. However, there is a paucity of research into interventions to manage obesity and weight gain in pregnancy [11,18,19]. An intervention programme entitled 'Eat Well Keep Active' aimed at increasing women's self-determination to eat a healthy diet and to exercise appropriately is proposed, that will incorporate SDT's three fundamental components.

This study aims to evaluate the feasibility and acceptability of the 'Eat Well Keep Active' intervention programme. Objectives of the study are threefold:

1. To conduct a small exploratory study of an intervention programme using motivational interviewing and goal setting entitled 'Eat Well Keep Active'.

2. To explore the feasibility and acceptability of the intervention.

3. To collect some preliminary qualitative data of the efficacy of the intervention at improving women's diet and exercise patterns.

## Methods/design

This is a small prospective study, using mixed methods to evaluate the feasibility and acceptability of an intervention programme delivered to 20 women early in their pregnancy. Pregnant women attending an antenatal clinic at a hospital in South West Wales for their first appointment will be approached to participate in the study. Those who are less than 15 weeks gestation and identified as being suitable for midwifery led care will be asked to participate. By only including women who are assigned to midwifery led care, participants will have no identified underlying health conditions or complications that may make them unsuitable for inclusion in the study. Women will be excluded from the study if they: are deemed to require consultant led care; are not fluent in English; are aged under 18 years; have a history of early pregnancy complications (e.g. vaginal bleeding) or a history of eating disorders. An estimated sample size of twenty has been chosen as it is felt that this will provide an adequate amount of data regarding the acceptability of the intervention.

The 'Eat Well Keep Active' intervention programme will be conducted by the lead author and comprises three aspects;

• a brief guiding session incorporating motivational interviewing and goal setting

• a personalised goal card

• a follow-up telephone call

Directly prior to the initial guiding session, a survey will be administered to assess diet quality and current daily activity levels [see Additional file 1]. Incorporated into the survey will be a set of questionnaires assessing two Self Determination Theory constructs: self-regulation, and perceived competence [20]. This will provide baseline data prior to the intervention.

### Recruitment

Prospective participants meeting the inclusion criteria will be identified by the antenatal clinic midwife through antenatal booking notes, and will be asked whether they wish to take part in the study. Potential participants will be contacted by telephone a week later allowing ample time to fully consider whether to participate or not. Women will be given the opportunity to ask questions of the researcher and written consent will be obtained prior to the delivery of the intervention. A suitable date and time will be arranged for the initial session with those who consent to take part.

#### Description of the 'eat well keep active' programme (see Figure 1)

**Figure 1:**
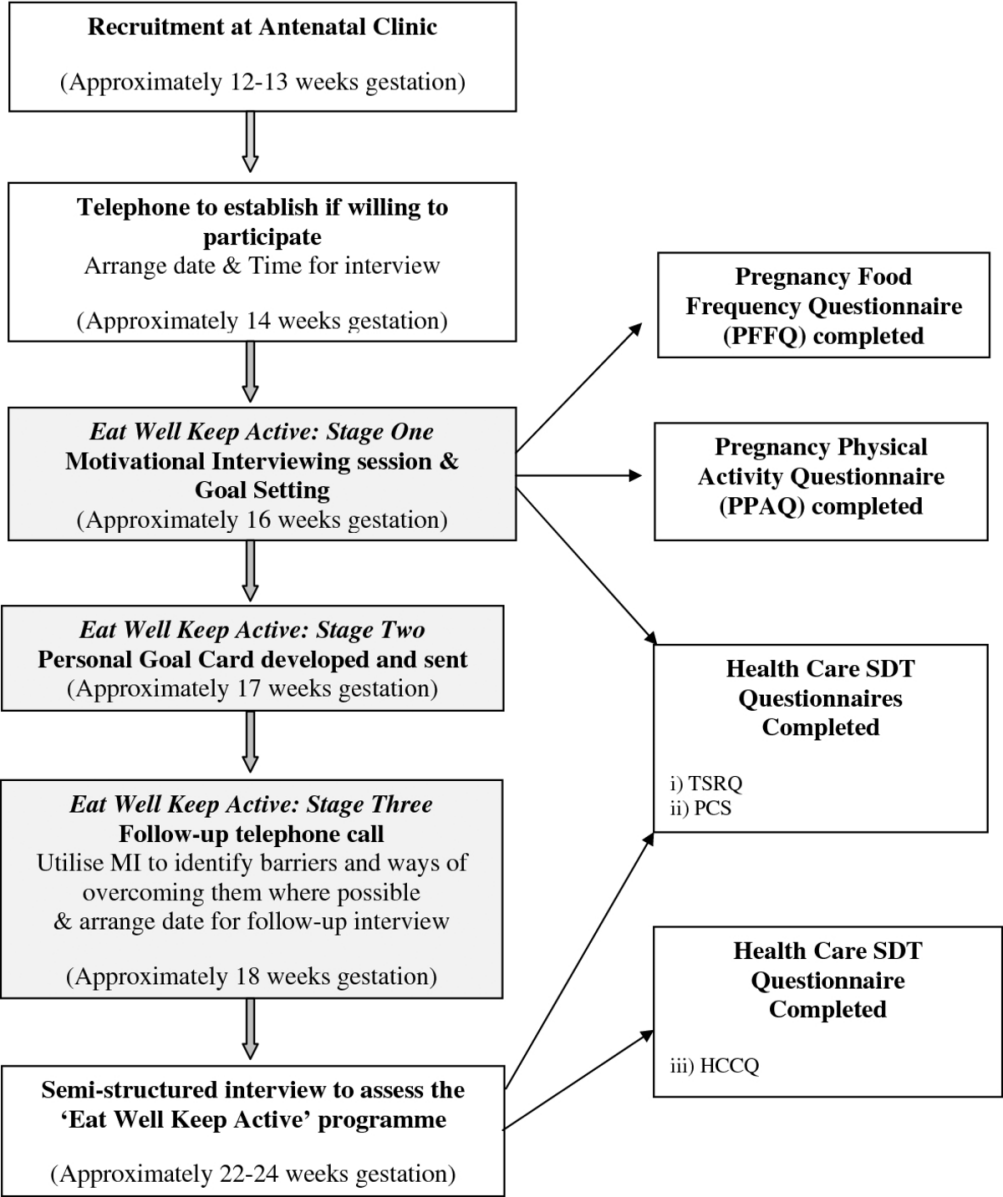
**Procedure flow-chart**.

#### Stage 1. Motivational interviewing and goal setting

**Figure 2:**
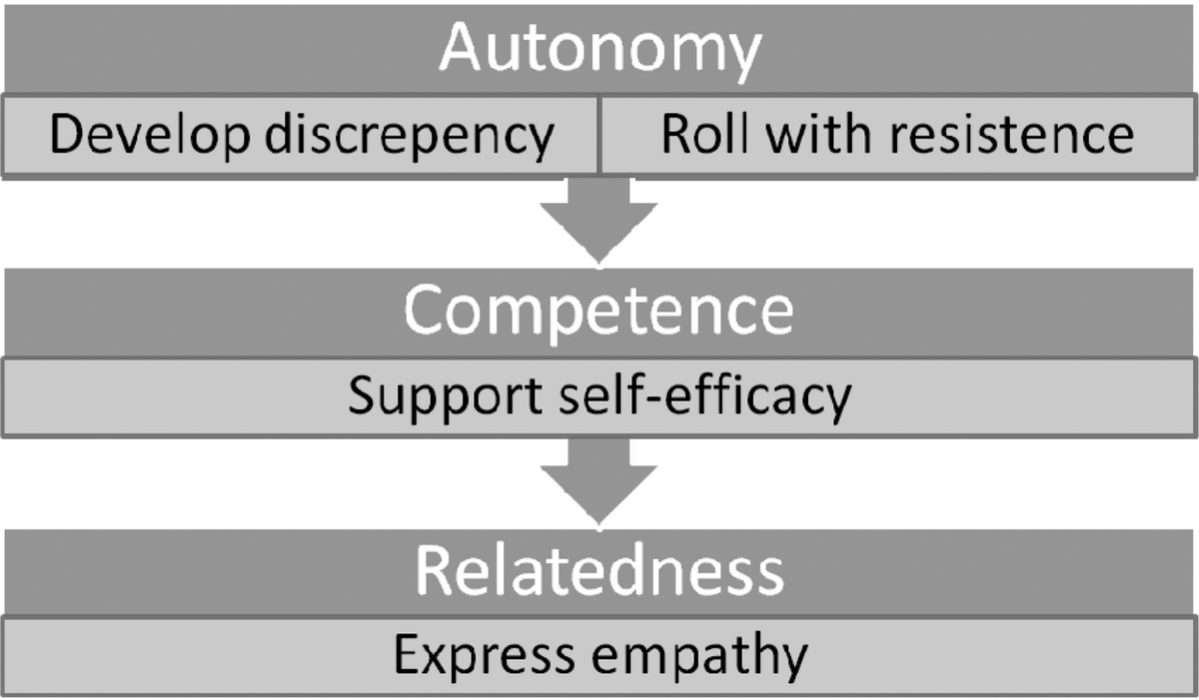
**Combining 3 self determination theory constructs with motivational interviewing principles**.

The first aspect of the programme will be a brief guiding session with the researcher incorporating Motivational Interviewing and goal setting. Motivational Interviewing (MI) is an approach developed by Miller and Rollnick [21] and is a directive client-centred technique aimed at eliciting behavioural change by exploring and resolving ambivalence. MI has been used widely and found to be an effective and robust method for facilitating behaviour change in a variety of behavioural domains [22,23]. Its client-centred approach reinforces personal responsibility and supports self efficacy. The researcher is trained in MI, and will use reflection throughout to ensure the 'spirit of MI' is adhered to. The guiding session will be based on the principles within Motivational Interviewing; express empathy, develop discrepancy, roll with resistance and support self-efficacy [21]. The social-environmental factors of support for autonomy, competence and relatedness identified in Self Determination Theory have clear parallels to the principles embedded within MI [23], and as such provide the theoretical underpinning to understand its processes and efficiency (see Figure 2).

Towards the end of the motivational interviewing session a proforma goal setting chart will be completed to assist in the development of the personalised goal card. This chart has been adapted for pregnant women from a tool used in behaviour change [24]. The chart includes illustrations on a range of health behaviours as well as some blank spaces. The inclusion of blank spaces ensures that the chart can be used to explore the areas which the individual feels are most important to them and is thus a client-centred agenda setting exercise. These will then later be used to develop the personalised goal cards for each participant. Goal setting as a strategy for behaviour change has been used widely across many disciplines including health, and research indicates that it can be an effective tool [25,26].

#### Stage 2: Personalised goal card

The second aspect to the 'Eat Well Keep Active' programme will be a personalised goal card which will be developed by the researcher following the guiding session and sent to the participant within a week. This card will detail individual goals identified during the initial session (such as limiting unhealthy food intake and incorporating gentle exercise, identifying barriers to achieving those goals and means of overcoming them). This goal card will support the MI and provide participants with a personalised individual record of the session that can be referred to. Each goal card will be unique to the participant.

#### Stage 3: Follow-up call

The third and final aspect of the 'Eat Well Keep Active' programme will be a follow-up telephone call two weeks after the first session that will again use Motivational Interviewing. The aim of the call is to offer support, assess the goal setting and to identify any unforeseen barriers that may impede achievement of goals. It is envisaged that using MI it will be possible to develop skills to overcome these barriers where viable.

### Outcomes

The primary outcome measures will be women's views on the acceptability and efficacy of the 'Eat Well Keep Active' programme. This will be obtained through one-to-one interviews 6-8 weeks following the delivery of the first guiding session. Secondary outcome measures include comparisons of the assessment of the Self Determination Theory constructs associated with behaviour prior to and following the delivery of the intervention. This information will be collected from the HC-SDT pack of questionnaires [20].

#### Women's views on the 'eat well keep active' programme

Semi-structured interviews will be used to evaluate the acceptability and participant perception of efficacy of the intervention. An interview schedule will be used to ensure the interview remains focused but the decision to use semi-structured interviews will provide the opportunity for further exploration providing richer data. The purpose of the interview is to gain an in-depth understanding of women's experiences of the intervention, including whether and why they found it helpful or not.

#### Pregnancy food frequency questionnaire (PFFQ)

The PFFQ was developed for the Avon Longitudinal Study of Pregnancy and Childhood (ALSPAC) for the purpose of analysing the dietary intakes of 11,923 pregnant women [27]. Subsequent studies using the PFFQ have found it to be a useful and reliable tool in the collection of dietary data of pregnant women [28]. This questionnaire has been adapted and revised as questions relating to consumption of food/drinks which are now contraindicated in pregnancy (e.g. alcohol, shellfish, liver) were removed from the questionnaire. Also questions pertaining to body image were removed, as this was not felt to be relevant to the current study.

#### Pregnancy physical activity questionnaire (PPAQ)

The PPAQ developed by Chasen-Taber and colleagues in America is a self-report instrument for measuring the time spent participating in activities including household chores, work related activity, sport and exercise as well as transport and sedentary behaviours [29]. The PPAQ has been assessed as being a valid and reliable measure of exercise and activity in pregnancy [29,30]. The PPAQ was modified for this study as the questionnaire repeatedly asks respondents about their activity levels 'During this trimester'. The word trimester was felt to be medical terminology that some may not understand. Instead the questions were altered to: 'During the last 4 weeks'.

#### Health care self determination theory pack (HC-SDT)

The HC-SDT is a package combining three brief questionnaires [20]. The first is the Treatment Self-Regulation Questionnaire (TSRQ) a 30 item measure which is used to assess an individual's motivation in terms of their degree of autonomy and self-regulation. The second is the Perceived Competence Scale (PCS) an 8-item measure which is used evaluate individuals' confidence to maintain healthy behaviour (diet and exercise). The third and final is the Health Care Climate Questionnaire (HCCQ), a 12-item measure used to assess respondents' perception of the degree to which they find their health-care provider autonomy supportive or controlling. This final questionnaire included in the HC-SDT pack will be used at the final evaluation meeting only, to assess the perception how autonomy supportive or controlling they have found the 'Eat Well Keep Active' programme of interventions delivered by the researcher. All three have been assessed as valid and have been widely used in the study of behaviour change in health care settings [20,31].

### Data collection

Data will be collected from participants at two time points (see Figure 1). Firstly, immediately prior to the guiding session participants will be asked to complete a questionnaire. The questionnaire contains the Pregnancy Food Frequency Questionnaire (PFFQ) and the Pregnancy Physical Activity Questionnaire (PPAQ). It is also proposed that participants will complete the Health Care Self Determination Theory package (HC-SDT) that relates to their diet and activity.

The second time point for data collection will be approximately 6 weeks after delivery of the start of the intervention programme, when participants will again be asked to complete the Health care SDT (HC-SDT) package which at this time point will include the Health Care Climate Questionnaire (HCCQ). They will also take part in a one to one semi-structured interview to assess the acceptability and perceived efficacy of the intervention. This interview will be digitally recorded to allow for transcription for later analysis.

### Data analysis

Mixed methods of data collection will be used to obtain information relating to acceptability and efficacy of the intervention under study. It is envisaged that changes in the degree of self-determination as a result of the intervention will be captured through the responses to the HC-SDT package. Quantitative analysis will be primarily descriptive in nature and will be displayed as frequencies. Appropriate tests will be applied to assess differences between pre and post intervention outcomes obtained from the responses given to the HC-SDT set of questionnaires. If the data meet the basic assumptions for parametric analysis then paired sample t-tests will be used. However, if the assumptions are not met, which is possible with such a moderate sample size, the non-parametric equivalent test, Wilcoxon will be used.

The data gathered from the semi structured interviews will be carefully transcribed and anonymised and will then be subjected to thematic analysis utilising NVivo (computer assisted qualitative data software) to identify commonly occurring themes and the relationships between them.

### Ethics

This study has been reviewed by the South West Wales Research Ethics Committee and has received a favourable ethical opinion. (11/WA/0018)

## Discussion

Acceptable and effective ways to assist women to eat a healthy balanced diet and to undertake appropriate levels of activity during pregnancy are needed to improve the health of the pregnant population [11] and may help to reduce the incidence of obesity following childbirth. This research is intended to establish whether the 'Eat Well Keep Active' programme of interventions is acceptable to women and to assess whether participants find it effective in improving diet and activity behaviours. This study is exploratory and the sample size is small, hence results regarding the efficacy of the intervention programme would need to be interpreted with caution. If results from this study suggest women have responded positively to this midwife-led intervention, it is planned to undertake a larger scale trial.

## Abbreviations

SDT: Self Determination Theory; MI: Motivational Interviewing; HC-SDT: Health Care Self Determination Theory pack; PFFQ: Pregnancy Food Frequency Questionnaire; PPAQ: Pregnancy Physical Activity Questionnaire; TSRQ: Treatment Self Regulation Questionnaire; PCS: Perceived Competence Scale; HCCQ: Health Care Climate Questionnaire.

## Competing interests

The authors declare that they have no competing interests.

## Authors' contributions

This study was originally conceived by LW, JR & BH. The 'Eat Well Keep Active' Programme was developed by LW with support from JR & BH. LW is responsible for data collection and originally drafted the study protocol. JR and BH supervised the project and made substantial contributions to the protocol. All authors read and corrected draft versions of the manuscript and approved the final manuscript.

## Authors' information

LW is a PhD student and is being supervised by JR & BH. All authors are based in the College of Human and Health Sciences, in Swansea University.

## Pre-publication history

The pre-publication history for this paper can be accessed here:

http://www.biomedcentral.com/1471-2393/12/27/prepub
